# Prevalence of Measles Antibodies in São José do Rio Preto, São Paulo, Brazil: A serological survey model

**DOI:** 10.1038/s41598-020-62151-3

**Published:** 2020-03-20

**Authors:** Cassia Fernanda Estofolete, Bruno Henrique Gonçalves de Aguiar Milhim, Carolina Cunha Galvão de França, Gislaine Celestino Dutra da Silva, Marcos Tayar Augusto, Ana Carolina Bernardes Terzian, Nathalia Zini, Edison Luís Durigon, Daniele Bruna Leal Oliveira, Eduardo Massad, Mauricio Lacerda Nogueira

**Affiliations:** 1 Laboratório de Pesquisas em Virologia, São José do Rio Preto Medical School (FAMERP), São José, São Paulo State Brazil; 2 Clinical and Molecular Virology Laboratory, Department of Biomedical Science, University of São Paulo (USP), São José, São Paulo State Brazil; 3 School of Applied Mathematics, Getúlio Vargas Foundation (FGV), Rio, Rio de Janeiro State Brazil

**Keywords:** Policy and public health in microbiology, Viral infection

## Abstract

Measles is an acute and highly contagious but vaccine-preventable infectious disease. Despite years of being considered eliminated, decreased vaccination rates have produced virus reemergence in several countries, including Brazil. Measles can be controlled through immunization programs, through which aim to achieve 95% coverage with two doses of the vaccine. Measles can also be controlled if suspected cases can be properly identified in order to contain outbreaks. This cross-sectional study determined the prevalence of measles antibodies and their correlation with rubella antibodies (resulting from the combination vaccine used in Brazil’s public immunization program) in individuals aged higher 10 years old in São José do Rio Preto, São Paulo State, Brazil, participants of a prospective cohort of arbovirosis surveillance before virus reemergence in the country. Our findings presented that 32.9% of individuals aged 10–40 years old had not antibodies against measles; 39.3% of total individuals with documented evidence of measles vaccination did not have anti-measles IgG, though only 20.2% of individuals with documented evidence of rubella vaccination lacked anti-rubella IgG. Besides, the most of measles cases reported in the city, following the virus spreading in the country, occurred especially in groups defined by us as susceptible. Because the combination MMR vaccine is part of Brazil’s national vaccine schedule, the possible reasons for this relatively high rate of seronegativity need to be investigated further, once that it reflects outbreak risk.

## Introduction

Measles is a highly contagious but vaccine-preventable disease. Its symptoms include acute rash, cough, and enanthem (including Koplik’s spots) on the oral mucosa^1,2^. Its most common complications include diarrhea, pneumonia, otitis media, laryngotracheobronchitis, and encephalitis, until deaths^3,4^.

In the prevaccine era, measles caused an estimated five to eight million deaths, and more than 90% of measles cases affected children under the age of ten^5,6^. The MMR (mumps, measles and rubella; being a manufacturer GlaxoSmithKline Biologicals, Belgium) vaccine has been offered to Brazilians since 1992, when it effectively replaced the monovalent measles vaccine^7^, but it is only since 2004 that it has been strongly and regularly recommended to infants turning one year old, followed by a second dose 3 months later^8^. The Brazilian National Immunization Program (BNIP) also provides non-previously inoculated teenagers and adults with the vaccine at any time.

As historic and current global numbers show, measles is one of the most contagious diseases. Its basic reproduction number (R-naught, or R0) is from 12 to 18, which means that each measles-infected person may spread the virus to 12 to 18 other individuals in a susceptible population^9^. To effective measles control, programs should achieve 95% coverage with two doses of the vaccine and promptly identify suspected cases in order to contain outbreaks^10^.

In 1998, Brazil began to experience a decline in measles rates as a result of its immunization program, and the last locally-acquired case of measles occurred in the year 2000^11^. However, an outbreak between 2013 and 2015 produced 1,310 reported cases. After the Pan American Health Organization implemented a plan of action, the endemic transmission of measles was considered eliminated in the Americas as of 2016^12^. But in 2017 four countries in the Americas confirmed cases of measles: Argentina, Canada, the United States, and Venezuela. Brazil has been experiencing a measles recirculation since 2018^13^. The current measles cases in the Americas has been caused mainly by genotype D8, which was first reported in Venezuela in 2017. Other countries in the region have reported both imported and imported-related cases of this genotype and lineage^13^.

Between epidemiological week (EW) 1 in 2018 and EW 31 in 2019 in Brazil, 11,371 measles cases were confirmed out of 22,654 suspected cases, 12 of which resulted in death^13^. From 2019 epidemiological week (EW) 1 to EW 31 in São Paulo State, 967 measles cases were confirmed out of 4,138 suspected cases; there were no deaths, and the most highly affected age group (45% of cases) was that of individuals between 15 and 29 years of age (45%)^14^. The city of São José do Rio Preto, located in northwest region of São Paulo State, Brazil, is considered an endemic area for dengue (DENV) and yellow fever virus (YFV) circulation^15,16^, and an important commercial hub with substantial human movement. Other arboviruses have been detected in this region, including Saint Louis encephalitis virus (SLEV)^17^, Zika virus (ZIKV)^18^, and coinfections therein^19^. Because of the city’s epidemiological history of these infections, the local government has established an ongoing cohort study to survey dengue and other arboviruses. In 2019, following the measles spreading in the country, 74 measles-confirmed cases were reported in the city (<6 months: 1; 6–11 months: 21; 1–5 years: 31; 6–29 years: 13; 30–59 years: 8 cases)^20^. Measles coverage in the city was reported as 103.3% (first dose) and 101% (second dose) in 2018^21^. Coverage in São Paulo State as a whole has been found to be 90.3% (first dose) and 81.3% (second dose), while coverage across Brazil has been determined to be 90.92% (first dose) and 75.72% (second dose)^22^.

Given the current global measles outbreaks and the threat of the continued spread of the disease, this study used data provided by an ongoing arbovirosis surveillance cohort to determine the prevalence of measles antibodies and to correlate the findings with demographic characteristics and data on immunization coverage in order to predict the risk of a local outbreak. The main objective of this study was described the population immunity status to measles in an epidemiological context before virus spreading in the city.

## Results

**Figure 1:**
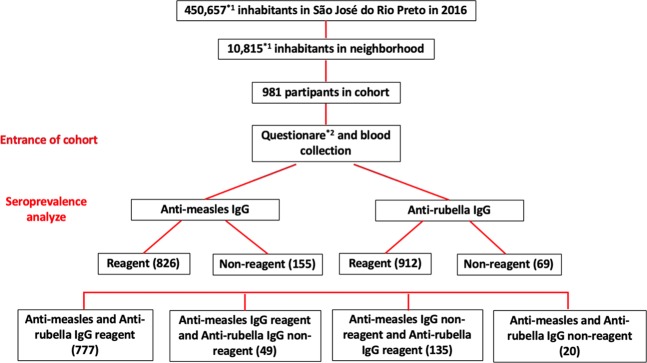
Prevalence of measles and rubella antibodies in the city of São José do Rio Preto, São Paulo, Brazil, 2016. *1: estimated population according to the São José do Rio Preto Health Secretariat 2016 report using 2015 metrics^31^. *2: including previous immunization (documented history of vaccination).

Among the 981 individuals tested to determine the presence of anti-measles IgG and anti-rubella IgG, seropositivity was found to be 84.2% (826/981) and 92.9% (912/981), respectively (Fig. 1). Measles and rubella seroprevalence results were organized by age group and demographic data, and the findings are presented in Tables 1 and 2. There was no significant difference in the seroprevalence results of measles or rubella between genders or ethnicities.

**Table 1:** Distribution of measles seroprevalence organized by age group and demographic data in the city of São José do Rio Preto, São Paulo, Brazil, 2016.

	Anti-measles IgG			
	Reagent (%)	Non-reagent (%)	*p*-value	OR (95% CI)
**Age range**				
10–20 years (n = 140)	86 (61.43%)	54 (38.57%)	—	1
21–30 years (n = 154)	98 (63.64%)	56 (36.36%)	0.7871	1.09 (0.68–1.76)
31–40 years (n = 141)	108 (76.6%)	33 (23.4%)	0.0088*	2.05 (1.22–3.44)
41–50 years (n = 180)	169 (93.89%)	11 (6.11%)	<0.0001*	9.64 (4.79–19.39)
>51 years (n = 365)	364 (99.73%)	1 (0.27%)	<0.0001*	228.55 (31.18–1,675–3)
**Gender**				
Male (n = 403)	339 (84.12%)	64 (15.88%)	0.9752	1.01 (0.71–1.43)
Female (n = 578)	487 (84.25%)	91 (15.75%)	—	
**Ethnicity**				
Caucasian (n = 421)	364 (86.46%)	57 (13.54%)	0.8285	1.07 (0.7–1.63)
Non-Caucasian (n = 313)	268 (85.62%)	45 (14.38%)	—	
**Marital Status**				
Married (n = 121)	101 (83.47%)	20 (16.53%)	0.045*	0.51 (0.27–0.94)
Not married (n = 129)	93 (73.1%)	36 (27.9%)	—	
**Place of Residence**				
Rural (n = 170)	147 (86.47%)	23 (13.53%)	0.4371	1.24 (0.77–2)
Urban and periurban (n = 811)	679 (83.72%)	132 (16.28%)	—	
**Schooling**				
8 years or less (n = 554)	507 (91.52%)	47 (8.48%)	<0.0001*	3.72 (2.56–5.4)
More than 8 years (n = 409)	304 (74.33%)	105 (25.67%)	—	
**Received Home Visit from Public Health Worker?**				
Yes (n = 870)	741 (85.17%)	129 (14.83%)	0.0667	1.73 (1–2.99)
No (n = 82)	63 (76.83%)	19 (23.17%)	—	
**Previously Diagnosed with a Chronic Disease**				
Yes (n = 339)	324 (95.58%)	15 (4.42%)	<0.0001*	6.0 93.4–10.41)
No (n = 621)	486 (78.26%)	135 (21.74%)	—	

*p < 0.05: statically significant.

OD: odds ratio; CI: confidence interval.

**Table 2:** Distribution of rubella seroprevalence organized by age group and demographic data in the city of São José do Rio Preto, São Paulo, Brazil, 2016.

	Anti-rubella IgG			
	Reagent (%)	Non-reagent (%)	*p*-value	OR (CI 95%)
**Age range**				
10–20 years (n = 140)	123 (87.86%)	17 (12.14%)	—	1
21–30 years (n = 154)	137 (88.96%)	17 (11.04%)	0.91	1.11 (0.54–2.27)
31–40 years (n = 141)	138 (97.87%)	3 (2.13%)	0.0024*	6.35 (1.82–22.21)
41–50 years (n = 180)	170 (94.44%)	10 (5.56%)	0.0574	2.35 (1.04–5.30)
>51 years (n = 365)	343 (93.97%)	22 (6.03%)	0.0181*	2.37 (1.20–4.67)
**Gender**				
Male (n = 403)	370 (91.81%)	33 (8.19%)	0.2917	1.34 (0.82–2.19)
Female (n = 578)	542 (93.77%)	36 (6.23%)	—	
**Ethnicity**				
Caucasian (n = 421)	396 (94.06%)	25 (5.94%)	0.436	1.31 (0.73–2.35)
Non-Caucasian (n = 313)	289 (92.33%)	24 (7.67%)	—	
**Marital Status**				
Married (n = 121)	117 (96.69%)	4 (3.31%)	0.4387	1.93 (0.56–6.59)
Not married (n = 129)	121 (93.8%)	8 (6.2%)	—	
**Place of Residence**				
Rural (n = 170)	156 (91.76%)	14 (8.24%)	0.6108	0.81 (0.44–1.49)
Urban and periurban (n = 811)	756 (93.22%)	55 (6.78%)	—	
**Schooling**				
8 years or less (n = 554)	520 (93.86%)	34 (6.14%)	0.1892	1.43 (0.87–2.33)
More than 8 years (n = 409)	374 (91.44%)	35 (8.56%)	—	
**Received Home Visit from Public Health Worker?**				
Yes (n = 870)	809 (92.99%)	61 (7.01%)	0.933	0.86 (0.33–2.2)
No (n = 82)	77 (93.9%)	5 (6.1%)	—	
**Previously Diagnosed with a Chronic Disease**				
Yes (n = 339)	319 (94.1%)	21 (5.9%)	0.5009	1.24 (0.73–2.11)
No (n = 621)	574 (92.43%)	47 (7.57%)	—	

*p < 0.05: statically significant.

OD: odds ratio; CI: confidence interval.

The highest anti-measles IgG seroprevalence rates were found among individuals over 40 years of age (533/545; 97.8%), while 32.9% (143/435) of individuals between 10 and 40 years of age were found to have no measles antibodies. In the case of rubella, individuals older than 30 years of age (651/686; 94.9%) were found to have higher seroprevalence than those between 10 and 30 years of age (260/294; 88.4%). Individuals in the subcohort between 10 and 20 years of age represent Brazilians who were infants and children when the Brazilian immunization program included the MMR vaccine and who were therefore vaccinated and likely not seroconverted after natural infection. In subjects in this age group, 61.43% were found to have measles antibodies, and 87.86% were found to have rubella antibodies, showing a significant part of population susceptible to natural infection to rubella, but mainly measles.

**Figure 2:**
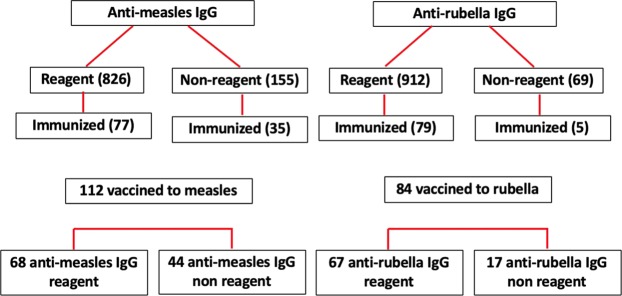
Correlations between documented history of vaccination and measles and rubella antibodies in the city of São José do Rio Preto, São Paulo, Brazil, 2016.

Data on subjects’ vaccine histories was obtained from the interpretation of subjects’ immunization booklets. Out of the 981 subjects included herein, 112 (112/981; 11.4%) had written proof of having received the measles vaccine (vaccination booklets), and 68 (60.7%; 68/112) were reagent to measles. In the case of rubella, 84 individuals (8.6%; 84/981) had written proof of having received the rubella vaccine (vaccination booklets), and 67 individuals (79.8%; 67/84) were reagent to rubella (Fig. 2). When seropositivity for measles and rubella was analyzed simultaneously, 4 of the 20 individuals (20%; 4/20) who presented neither anti-measles IgG nor anti-rubella IgG had been vaccinated. All 4 of these individuals were between 10 and 30 years of age.

**Figure 3:**
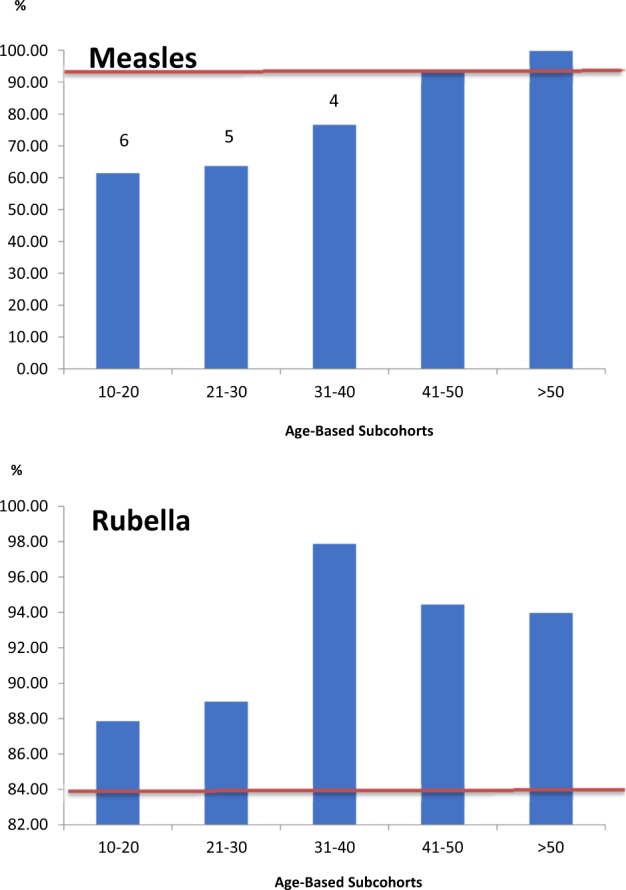
Estimates for each infection that presented antibody prevalence above the herd immunity threshold* and the number of secondary cases that would occur in each age cohort if a single infectious individual had contact with the remaining susceptible individuals. *Basic reproduction numbers for measles and rubella assumed to be 15 and 6, respectively 9. Red line: herd immunity threshold.

Analyzing antibody prevalence in the context of the basic reproduction numbers for measles and rubella, only the subcohort above 40 years of age can be considered to be effectively protected against measles, while all age group was considered protected against rubella (Fig. 3). The remaining subcohorts (10–40 years) were found to be susceptible to measles; the expected numbers of secondary cases produced if a single infectious individual were introduced into the community were 6 for the subcohort between 10 and 20 years of age, 5 for the subcohort between 21 and 30 years of age, and 4 for the subcohort between 31 and 40 years of age (Fig. 3).

## Discussion

This serological surveillance study has helped to identify the proportion of individuals susceptible to measles in a single, city-wide population in Brazil during a time in which the country is experiencing an outbreak. The highest measles seroprevalence rate was found in the subcohort older than 40 years if age. This finding was expected, as older individuals have more chances of exposure to measles and vaccines as they age. However, the most remarkable finding was the high rate of non-reagent individuals between 10 and 40 years of age (32.9%), a result which shows that this subcohort is susceptible to infection. Besides, 39.3% and 20.2% had proven documentation of measles and rubella, respectively, but not serological evidence (non-reagent antibodies to measles and rubella). The relatively high potential for measles to spread is supported by the expected numbers of secondary cases in the subcohorts younger than 40 years of age.

The measles virus has one of the highest contagion indices among known human pathogens^9^. Two doses of the MMR vaccine are required to reach immunity thresholds and terminate transmission^23^. According to World Health Organization (WHO) recommendations^4^, to decrease the incidence of measles and lengthen the intervals between outbreaks (e.g., 4–8 years) relative to those observed during the prevaccine era (e.g., 2–4 years), more than 80% of individuals should have antibodies to measles, a rate which was observed herein only among individuals older than 40 years of age. Given the inclusion of two doses of the measles vaccine in the Brazilian government’s official immunization schedule^8^, such low rates of anti-measles IgG was not expected in the younger subcohorts, particularly those which were targets of vaccination campaigns^7^. The spread of vaccine refusal has become a risk factor for measles outbreaks, and the WHO has identified vaccine hesitancy as one of the top ten global health threats in 2019^24^.

It is also concerning that 32.9% of individuals between 10 and 40 years of age lacked anti-measles IgG, some of whom had been vaccinated. Serological and epidemiological evidence indicates that measles-containing vaccines provide long-lasting immunity to most individuals. Approximately 95% of those vaccinated have been found to have detectable measles antibodies 11 years after receiving the initial MMR vaccination and 15 years after receiving the second dose^23^. Another study on subjects in India between 18 and 23 years of age found rates of seropositivity of 79%, 91%, and 88% for measles, mumps, and rubella, respectively, as well as a higher overall seroprevalence among males^25^. In a study on subjects in the United States between 12 and 29 years of age, the rates of measles, mumps, and rubella seropositivity were found to be 92.0%, 87.6%, and 95.3%, respectively^26^.

Among our subjects in this age group, the finding of 67.1% anti-mesles seroprevalence is lower than the rates reported in the literature. Only subjects older than 40 years of age exhibited effective protection against measles in the study population. The individuals in this subcohort were born before the introduction of the measles vaccine in Brazil and therefore likely tested positive to measles antibodies through natural infection or contact with the virus. The remaining subcohorts were considered to be susceptible to measles, since vaccination coverage was insufficient to reach the heard immunity threshold of 93.3%. Such susceptibility was noted through measles cases number occurred in 2019. Among 74 reported cases, more then 60% of them were in individuals aged lower than 30 years^20^.

Among the subjects who had documented proof of the measles vaccine, only 60.7% were found to be reagent. Meanwhile, 79.8% of the subjects with documented proof of rubella immunization were found to be reagent. Seroprevalence after measles, mumps, and rubella vaccinations has been estimated to be 88%, 97%, and 97%, respectively^26^. Almost all individuals who fail to respond to the measles component of the first dose of the MMR vaccine received after one year of age respond to the second dose^23^. For this reason, immunization programs, including the Brazilian immunization schedule^8^, recommend at least two doses in children and adolescents.

Another study found the measles vaccine to be highly effective after a single dose when it is administered after one year of age, with a median effectiveness of 93% (range: 39–100%); effectiveness was found to be even higher after two doses, with a median rate of 97% (range: 67–100%)^24^. Furthermore, many individuals who lack a detectable antibody develop a secondary immune response upon revaccination or exposure to the wild-type virus, a phenomenon which suggests the persistence of some level of immunity^27^.

So, when compared to researchers’ findings, the results on rubella immunity obtained herein are lower than but consistent with those; however, the findings on measles immunity in our population vary substantially from those of other studies and there is an important difference between measles and rubella immunization in our population in each age group. The quality and durability of measles vaccine-induced immunity is dependent upon a number of factors involving both the vaccine used, including inappropriate use, storing, or delivery of vaccines, and the host, In this study, all 35 subjects who had been vaccinated against measles but who tested non-reagent were younger than 40, and only 2 had a chronic disease (one case of cognitive deficits and one case of vitiligo; neither used immunosuppressive drugs).

It is also important to distinguish between primary vaccine failure, which is the failure to seroconvert after vaccination, and secondary vaccine failure, which is the loss of protection after seroconversion has been demonstrated. Vaccine-induced immunity (secondary vaccine failure) wanes in 0% to 6% of vaccinations, but this phenomenon does not seem to play a major role in measles virus transmission or in reducing a given population’s overall immunity to measles^27,28^. In this study, 39.3% of individuals with documented evidence of measles vaccination did not have anti-measles IgG, while 20.2% of individuals with documented evidence of rubella vaccination lacked anti-rubella IgG. When we observed our data, if we analyze only group 10–20 years (n = 140), those vaccine with MMR (combined vaccine), 36 of them had measles and rubella vaccine report, being the 36 reagent to anti-rubella IgG and 24 to anti-measles IgG. Because most of these subjects were likely exposed to the viruses through the MMR vaccine rather than natural infection, the differences between the measles and rubella seroconversion rates seem to be the result of the components of the vaccine.

The main limitation of this study was that it was cross-sectional in nature. Our primary objective with populational cohort was performing an arbovirosis surveillance. However, with measles reemergence in the country, our samples might be used to contribute to know the serological status of immunization in our population. Besides, in clinical trials, control subjects are healthy and are specifically selected, and vaccines are stored and administered according to strict protocols—protocols which are often not put into practice in routine field or clinical vaccination^29^. These findings represent a real epidemiological context in a population in surveillance. Another limitation of this study was the subjects’ lack of documentation regarding their immunization history, founded in few cases.

The findings obtained herein reflect the urgent need for a re-evaluation of Brazil’s current immunization program and the population’s compliance with it. Seroprevalence surveillance allows for the identification of subgroups with higher susceptibility, and the findings may be used to guide immunization policies. We believe that the seronegativity rates observed in our study reflect the need for action, since measles is highly transmissible and requires very high immunity rates^29^. These results raise questions about the efficacy of measles immunization in these individuals and, consequently, in the general population.

Although these findings are not necessarily representative of measles seroprevalence in Brazil as a whole, they are representative in the city once to reflect demographic profile and health care model used in other neighborhoods. As it was observed, the understanding of the dynamic of antibodies in the population may highlight to susceptibility profiles. So, particularly for diseases that have an impact on global health, the need for larger-scale serological studies in order to better to know the results of immunization program beyond the vaccine coverage.

## Methods

### Study design

This was a descriptive cross-sectional study performed on a prospective arbovirus surveillance cohort. The study included subjects 10 years of age or older who were residents of the study area (a single neighborhood in the city of São José do Rio Preto, representative of population due to demographic and spatial characteristics observed in municipal monitoring panel^30,31^) and who had been previously included in the cohort. Informed consent was obtained from all adult subjects and from the parents and/or legal guardians of subjects under 18 years of age.

This study and the larger cohort surveillance study have been approved by the Ethical Review Board from Faculdade de Medicina de São José do Rio Preto (Comissāo de Ética em Pesquisa em Seres Humanos da FAMERP – CEP/FAMERP) (ERB number 02078812.8.0000.5415). All performed experiments in this study are according with relevant protocols and also approved by institutional ERB. Confidentiality was ensured by the encoding of questionnaires and samples before data entry and analysis.

### Sample and data collection

A total of 981 individuals were included in this study. When they were originally included in the cohort, in 2016 (before measles spreading in the country as reemergent virus responsible to recent outbreak), all individuals had blood samples collected for the detection of anti-measles and anti-rubella Immunoglobulin G (IgG). They also completed a questionnaire about demographic characteristics. Data on vaccination history were also collected from vaccination booklets from 112 individuals.

### Sample analysis

Collected sera were frozen and later tested using a commercial enzyme-linked immunosorbent assay (ELISA Euroimmun AG, Lubeck, Germany). Tests were used for the detection and qualitative measurement of IgG antibodies to measles and rubella following the manufacturer’s instructions. IgG antibodies to measles or rubella were categorized based on index standard ratio (ISR) values: seronegative was defined as an ISR less than 0.8; indeterminate results were defined as an ISR between 0.8 and 1.1; and seropositive was defined as an ISR greater than 1.1. Indeterminate was considered as negative to final analyses.

### Statistical analysis

The individuals included in this study were randomly selected from the population and then organized into age-based subcohorts with subjects 10 to 20 years of age (born between 1996 and 2006), 21–30 years of age (born between 1995 and 1986), 31 to 40 years of age (born between 1985 and 1976), 41 to 50 years of age (born between 1975 and 1966), and older than 50 (born in 1965 or earlier). As mentioned previously, vaccination against measles in Brazil was introduced in 1967 as a single-dose schedule. This was changed to a two-dose schedule in 1992, and the MMR vaccine became part of the routine schedule in 2004. The age-based subcohort that included subjects older than 50 therefore included unvaccinated individuals who were born in the prevaccine era, while the subcohorts that included subjects between 21 and 40 years of age included both vaccinated and unvaccinated individuals. The subcohorts with subjects younger than 20 years of age included only individuals who were born after the MMR vaccine became routine in Brazil and were therefore likely to have been seroconverted only after immunization rather than natural infection. Because of the lack of previous studies on seroconversion after natural infection or immunization and of studies with data organized by age, which would be required to estimate the expected prevalence rate of antibodies, we considered the known population (10,815 individuals in the study area) and a tolerable sample error of 4% to establish the minimum sample number for this study.

The chi-squared test and Student’s t-test were used to compare the serological statuses of measles and rubella based on different characteristics. A *p*-value less than 0.05 was considered statistically significant. Using the age-dependent seroprevalence profiles for measles and rubella, we estimated a sample size that would present antibody prevalence above the herd immunity threshold for each virus. Basic reproduction numbers for measles and rubella were assumed to be 15 and 6, respectively^9^. Based on these numbers, we calculated the number of secondary cases that would be produced in each age-based subcohort if a single infected individual had contact with the remaining susceptible individuals.
